# Cost-Effectiveness Analysis of Different Genetic Testing Strategies for Lynch Syndrome in Taiwan

**DOI:** 10.1371/journal.pone.0160599

**Published:** 2016-08-02

**Authors:** Ying-Erh Chen, Sung-Shuo Kao, Ren-Hua Chung

**Affiliations:** 1 Department of Insurance, Tamkang University, Tamsui Dist., New Taipei City, 251, Taiwan; 2 Division of Gastroenterology, Department of Internal Medicine, Kaohsiung Veterans General Hospital, Kaohsiung, 813, Taiwan; 3 Institute of Population Health Sciences, National Health Research Institutes, Zhunan, 350, Taiwan; Ohio State University Medical Center, UNITED STATES

## Abstract

Patients with Lynch syndrome (LS) have a significantly increased risk of developing colorectal cancer (CRC) and other cancers. Genetic screening for LS among patients with newly diagnosed CRC aims to identify mutations in the disease-causing genes (i.e., the DNA mismatch repair genes) in the patients, to offer genetic testing for relatives of the patients with the mutations, and then to provide early prevention for the relatives with the mutations. Several genetic tests are available for LS, such as DNA sequencing for MMR genes and tumor testing using microsatellite instability and immunohistochemical analyses. Cost-effectiveness analyses of different genetic testing strategies for LS have been performed in several studies from different countries such as the US and Germany. However, a cost-effectiveness analysis for the testing has not yet been performed in Taiwan. In this study, we evaluated the cost-effectiveness of four genetic testing strategies for LS described in previous studies, while population-specific parameters, such as the mutation rates of the DNA mismatch repair genes and treatment costs for CRC in Taiwan, were used. The incremental cost-effectiveness ratios based on discounted life years gained due to genetic screening were calculated for the strategies relative to no screening and to the previous strategy. Using the World Health Organization standard, which was defined based on Taiwan’s Gross Domestic Product per capita, the strategy based on immunohistochemistry as a genetic test followed by BRAF mutation testing was considered to be highly cost-effective relative to no screening. Our probabilistic sensitivity analysis results also suggest that the strategy has a probability of 0.939 of being cost-effective relative to no screening based on the commonly used threshold of $50,000 to determine cost-effectiveness. To the best of our knowledge, this is the first cost-effectiveness analysis for evaluating different genetic testing strategies for LS in Taiwan. The results will be informative for the government when considering offering screening for LS in patients newly diagnosed with CRC.

## Introduction

Lynch syndrome (LS), also referred to as hereditary non-polyposis colorectal cancer (HNPCC), is an autosomal dominant disease caused by mutations in DNA mismatch repair (MMR) genes [1]. These genes include mutL homolog 1 (*MLH1*), mutS homolog 2 and 6 (*MSH2* and *MSH6*), and PMS1 homolog 2, mismatch repair system component (*PMS2*) [2, 3]. Patients with LS have increased risks of colorectal cancer (CRC) and other cancers, such as endometrial, ovarian, and stomach cancers, where the risk of CRC is the highest [4]. Based on a population-based cohorts from Europe and North America [5], it was estimated that LS accounts for 2.2% of patients who were newly diagnosed with CRC, making it the most common hereditary CRC predisposing syndrome.

Several genetic tests are available for LS, such as DNA sequencing for MMR genes and tumor testing using microsatellite instability (MSI) and immunohistochemical (IHC) analyses. The Evaluation of Genomic Application in Practice and Prevention (EGAPP) Working Group, sponsored by the Centers for Disease Control and Prevention (CDC) in the US, recommended offering genetic testing for LS to all patients who were newly diagnosed with CRC [6]. This universal screening for LS aims to identify mutations in patients newly diagnosed with CRC and then to provide testing and increased surveillance to their relatives with the mutations. Therefore, morbidity and mortality rates for CRC in the relatives can be reduced. Different screening strategies can be implemented by offering different genetic tests to patients newly diagnosed with CRC [7]. Cost-effectiveness analyses have been performed for different screening strategies and some strategies have been found to be cost-effective [8–12]. However, a recent study using German data reported that each of their evaluated screening strategies proved to be expensive [13], suggesting that findings from cost-effectiveness analyses for LS screening may vary in different countries.

In Taiwan, CRC has the second highest incidence rate among the top ten cancers, based on the 2012 Cancer Registry Annual Report (CRAR) released by the Taiwan government. The Ministry of Health and Welfare (MOHW) of the Taiwan government offers screening for CRC using fecal immunochemical testing (FIT) for people between 50 and 69 years of age every two years [14]. People with positive FIT results are further examined with colonoscopy. The prevalence of LS among newly diagnosed CRC patients in Taiwan was estimated as 2.3% [15]. However, the LS diagnostics are only available in some medical centers, such as the National Taiwan University Hospital. There are two main reasons for the limited availability of the LS diagnostics in Taiwan. First, colonoscopy is covered by the national health insurance provided by the National Health Insurance Administration (NHI) of the Taiwan government, while the LS diagnostics are not covered by NHI. Therefore, potential LS patients (e.g., relatives of CRC patients) would prefer colonoscopy over the self-paid LS diagnostics. The second reason is that the aforementioned FIT screening also reduces the potential LS patients’ motivation to adopt the LS diagnostics. Furthermore, a screening strategy for LS is not offered or recommended by the MOHW. This may be partly due to the fact that cost-effectiveness analysis for various screening strategies for LS has yet to be conducted in Taiwan. Hence, conducting a cost-effectiveness analysis for different screening strategies for LS from the MOHW’s perspective in Taiwan has become important.

Four screening strategies for LS were evaluated by the EGAPP [7] working group and were further investigated by Mvundura et al. [9]. The study by Mvundura et al. [9] concluded that some of the screening strategies can be cost-effective relative to no screening using a critical value of $50,000 or $100,000 per life-year (LY) saved. In a recent study, they used more conservative parameter values, which resulted in fewer LYs saved for the four screening strategies and the same conclusions can still be made [16]. However, because a cost-effectiveness analysis has not been conducted for the four screening strategies in Taiwan, it is unclear if any of these strategies will be cost-effective in Taiwan. Therefore, in this study, we conducted a cost-effectiveness analysis for the four LS screening strategies using the population-specific parameters in Taiwan. The analysis was performed from the MOHW’s perspective, which will be informative to the MOHW when they consider providing genetic screening for LS in Taiwan.

## Materials and Methods

### Review of the screening strategies for Lynch syndrome

**Fig 1:**
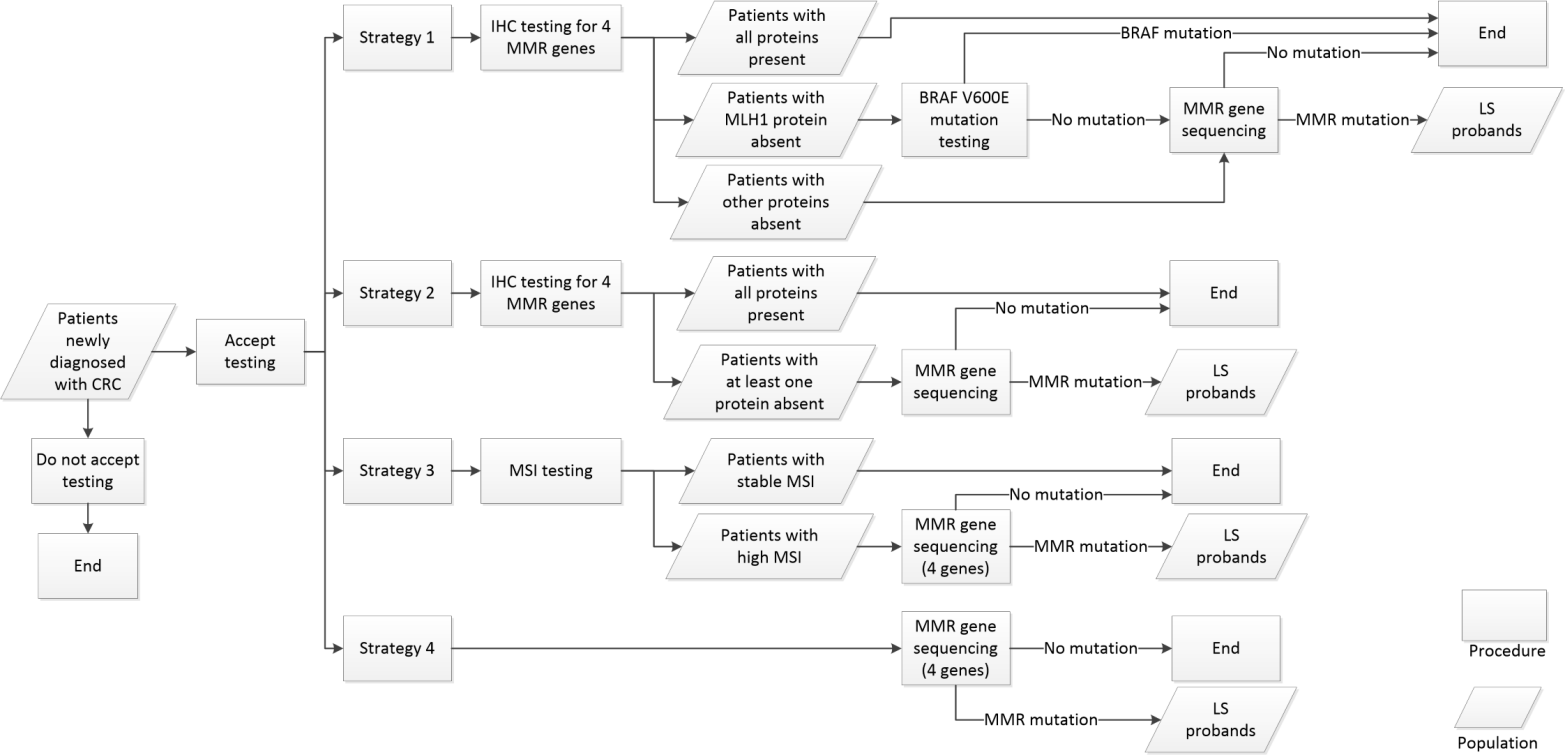
Flowchart for the four strategies evaluated in this study.

The four screening strategies considered in Mvundura et al. [9] were evaluated in this study. We briefly described the four strategies; more details can be found in Mvundura et al. [9]. A flowchart for the four strategies is shown in Fig 1. Patients newly diagnosed with CRC either accept or do not accept testing. Strategy 1 starts by offering IHC staining for the four MMR proteins to patients newly diagnosed with CRC. If the MLH1 protein stain is absent for a patient, the patient is tested for the BRAF V600E mutation. For a patient either with one of the three other protein stains absent or with negative BRAF V600E test result, the gene with the absent protein is sequenced in the patient. Strategy 2 also starts by offering IHC staining for the four MMR proteins; patients with an absent protein are then sequenced for the gene with the absent protein. Meanwhile, instead of the IHC test, Strategy 3 starts with MSI testing for the newly diagnosed patients with CRC. A patient with a high MSI result is sequenced for the four MMR genes. In Strategy 4, a patient with CRC is directly sequenced for the four MMR genes. Patients who were newly diagnosed with CRC confirmed with MMR mutations from one of the testing strategies are referred to as LS probands.

Some studies also included clinical criteria such as the Amsterdam II criteria and the revised Bethesda criteria in the screening strategies [10, 12, 13]. For example, only patients newly diagnosed with CRC who fulfill the clinical criteria were offered genetic testing. However, as argued by Palomaki et al. [7], a family history of CRC that is used in these criteria may not be reliable. Moreover, incorporating clinical criteria in the screening strategy can result in the reduced number of LS probands [12]. Furthermore, when excluding the strategies considering clinical criteria in Ladabaum et al. [10], Strategy 1 proved to be the most cost-effective strategy. Finally, although family histories are asked and recorded in medical charts in individual hospitals in Taiwan, the records are not shared among hospitals. Hence, a precise family history may be difficult to obtain for a CRC patient in Taiwan. Therefore, in this study, we focused on evaluating the effectiveness of the four strategies when applied to the Taiwan population excluding the clinical criteria.

Both universal testing for newly diagnosed CRC at all ages and age-targeted testing for newly diagnosed CRC < 50 years of age were considered in Mvundura et al. [9]. However, there were only about 1,700 newly diagnosed CRC with ages < 50 in Taiwan in 2012, resulting in fewer than 30 LS probands. Hence, we focused on evaluating universal testing in this study. A total of approximately 14,000 patients newly diagnosed with CRC were targeted in this study, according to the 2012 CRAR provided by the Taiwan government. Consistent with Mvundura et al. [9], we assumed that two thirds of the patients would accept testing after counseling—a more conservative rate when compared with the 90% and 85% rates used in Ladabaum et al. [10] and Severin et al. [13], respectively. Generally the patients in Taiwan are aware that CRC can be hereditary. The cost of genetic testing would be the major concern for the patients to adopt the testing. Therefore, if the cost is covered by the screening program, the assumption of the acceptance rate (i.e., 2/3) should be reasonable. First-degree relatives of LS probands would be contacted and offered genetic testing for the family-specific mutation. Similar to other studies [7, 9, 13], we assumed that an average of four relatives would be contacted and that half of them would accept genetic counseling and testing. This is a relatively conservative assumption as other studies assumed more relatives are contacted. For example, Ladabaum et al. [10] assumed eight people. Under a dominant inheritance model for LS, we assumed that 50% of the relatives would carry the family-specific mutations. The rate of uptake for genetic counseling among the relatives was assumed as 52%, and 95% of the relatives accepting counseling would accept genetic testing [7]. Relatives with confirmed MMR mutations from testing would be recommended for a colonoscopy every two years from the age of 20, and 79% of the relatives were assumed to adopt the increased CRC surveillance, the same proportion used in other studies [9, 10]. Relatives with confirmed LS not accepting the increased colonoscopic surveillance were assumed to undergo routine CRC screening (i.e., FIT every two years between 50 and 69 years of age followed by colonoscopy with positive FIT).

### Model parameters

We first described population-specific parameters for Taiwan. The CRAR published by the Taiwan government annually reports the numbers of patients newly diagnosed with cancers stratified by age, sex, clinical and pathological stages of the cancers, and types of treatments for the year. We obtained the latest CRAR, which was for the year 2012. A total of 13,534 newly diagnosed CRC patients were reported in the CRAR. The incidence rates of CRC by ages in the general population were calculated based on the numbers of CRC cases stratified by age groups in the CRAR and the 2012 census data of Taiwan stratified by age groups obtained from the Department of Household Registration of the Taiwan government. Based on a cohort of 5 million individuals [14], Chiu et al. estimated that the participation rate of FIT screening in Taiwan between the ages of 50 and 69 is 21.4%. The stage distributions of CRC at diagnosis without any surveillance and with FIT screening were also calculated using the same cohort by Chiu et al. [14]. The stage distributions in the population without surveillance were found to be 21.2%, 27.8%, 31.8%, and 19.2% for Stages 1, 2, 3, and 4, respectively, when compared with 40%, 36%, 19%, and 5% as calculated by Mvundura et al. [9]. Therefore, a significantly higher proportion of Stage 4 for CRC was observed in Taiwan than that in the US, which again addressed the importance of evaluating LS screening strategies in Taiwan. The stage distributions of CRC with FIT were 48.2%, 20.9%, 23.7%, and 7.2% for Stages 1, 2, 3, and 4, respectively. The stage distributions of CRC at diagnosis with the surveillance of colonoscopy every two years were adjusted based on the same method in Mvundura et al. [9], assuming that the surveillance resulted in 20% increase of the survival rate of CRC.

The five-year survival rates for CRC at different stages were obtained from a study based on 17,526 cohorts in Taiwan [17]. The prevalence of LS in newly diagnosed CRC patients in Taiwan was estimated to be 2.3% [15], similar to the estimate of 2.2% in Caucasians [5]. Mutations in *MLH1*, *MSH2*, *MSH6*, and *PMS2* have been reported in several studies based on Chinese cohorts with LS [18–21]. Table 1 shows the numbers of mutations in these four genes identified by these studies. The weighted proportions of LS with mutations in the four genes were calculated using the approach in Palomaki et al. [7], which assumed that LS was solely caused by the four genes. The proportions were estimated as 58%, 25%, 11%, and 6%, for *MLH1*, *MSH2*, *MSH6*, and *PMS2*, respectively, for the Chinese when compared with 32%, 39%, 14%, and 15% calculated in Palomaki et al. [7] for the Caucasians.

**Table 1:** The numbers of mutations in the MMR genes reported by studies based on the Chinese population.

	*MLH1*	*MSH2*	*MSH6*	*PMS2*	Sample size
Ni [18]	52	22	NA	NA	153
Sheng et al. [20]	8	4	0	NA	21
Yan et al. [19]	NA	NA	4	NA	39
Sheng et al. [21]	NA	NA	NA	1	26
Weighted proportion	58%	25%	11%	6%	

Some parameter values that have not been reported in the literature for Chinese were adopted from other studies based on Caucasians, assuming their values were not significantly different among different populations. For example, age-specific incidence rates of CRC in LS mutation carriers estimated from Bonadona et al. [4] were adopted for cost-effectiveness analyses by Grosse et al. [16] and Severin et al. [13]; the same data were also used in our study. The one-year risk of developing second CRC in LS mutation carriers was assumed to be 1.6% [22], the same value used in Mvundura et al. [9] and Severin et al. [13]. The age distribution of relatives with LS mutations was assumed to be normal, with a mean of 35 and standard deviation of 12, similar to the distribution used in Severin et al. [13]. We also assumed that the surveillance resulted in 59% reduction in risk of developing CRC in LS mutation carriers [23], a more conservative estimate used in Grosse et al. [16] than that (i.e., 62%) in Mvundura et al. [9]. Finally, the sensitivities and specificities for the genetic tests in the Taiwan population were assumed to be the same as those reported in the literature [7]. S1 Table shows the population-specific parameters in Taiwan as well as those adopted from other studies.

### Measuring costs

Most of the cost information was obtained from the website of NHI (http://www.nhi.gov.tw). The NHI provides national health insurance with a coverage rate of more than 99% of the Taiwan population. The NHI fee schedule includes the points reimbursed to the hospitals for the tests or treatments covered by NHI. Generally, one point is equal to 70% to 80% of one Taiwan New Dollar (TWD), depending on the budget of NHI for the year. We assumed the rate to be equal to 80% in our cost calculations. Genetic testing is generally not covered by the current national insurance plan provided by NHI. However, the NHI website also lists the costs of self-paid treatments and testing charged by 21 major hospitals in Taiwan. The costs differ among hospitals. As suggested by Mvundura et al. [9], the costs represent upper bound estimates because the actual costs reimbursed by NHI would be lower than the costs charged by the hospitals. All of our costs obtained in TWD were adjusted based on consumer price index (CPI) as 2012 and converted to US dollars. One TWD was exchanged as US $0.034 on average in 2012. All costs were discounted at 3%, the rate commonly assumed in cost-effectiveness analyses.

Lifetime treatment costs for CRC at different stages were also obtained from the study based on the 17,526 cohorts in Taiwan by Chen et al. [17]. Due to the limit of the survival model used in Chen et al. [17], costs for Stage 1 were not estimated; instead, we assumed that costs for Stage 1 were the same as those for Stage 2. Note that our assumption might overestimate the Stage 1 costs, because the treatment costs for Stage 2 are generally higher than that for Stage 1. Lifetime treatment costs for the second CRC have not been estimated in Taiwan. We assumed that the increased rate of costs for the second CRC compared with the costs for the first CRC was the same as that observed in Mvundura et al. [9] at each stage. Costs used in our analyses are also provided in S1 Table.

Following Mvundura et al. [9], three types of costs were calculated based on the MOHW’s perspective: the costs of detecting LS probands, the costs of detecting relatives with LS mutations, and costs of surveillance and treatment for CRC for the relatives. The costs of detecting LS probands included the costs of offering and performing the genetic testing, and the costs of genetic counseling before and after testing. The costs of detecting relatives with LS mutations included the costs of locating the relatives, the costs of genetic testing for the family-specific mutations, and the costs of genetic counseling before and after testing. The costs of surveillance and treatments for CRC for the relatives included the costs of colonoscopy every two years after the age of 20, the costs of treatments for the complications during colonoscopy, and the costs of lifetime treatments.

### Decision analytic model

Similar to other studies [10, 13], decision trees along with Markov models were used in our decision analytical modeling. A cohort of 13,534 individuals newly diagnosed with CRC was first simulated, where each individual had 4 relatives with a mean age of 35 years. Based on the prevalence of LS in patients newly diagnosed CRC in Taiwan, 2.3% of the CRC cohorts were assigned to be affected by LS. Among the relatives of such newly diagnosed CRC patients who had LS, 50% were LS mutation carriers. For those patients newly diagnosed with CRC but without LS, the LS status for the relatives of the patients was determined by the population prevalence of LS (i.e., 0.227% [24]).

Further, mutations in MMR genes in LS patients were generated based on the proportions of MMR mutations in LS carriers. Each patient newly diagnosed with CRC then entered the decision trees modeling the four screening strategies as well as the Referent strategy. The Referent strategy refers to the strategy where 21.4% of individuals with ages between 50 and 69 years undergo the FIT screening every two years, followed by colonoscopy if FIT is positive, while all other individuals are under no surveillance for CRC. Relatives with LS mutations subsequently entered the Markov model, and life expectancy for the relatives was estimated. Various parameters were incorporated in the Markov model, including the risks of developing CRC in LS carriers at different ages, the reduction of risk due to surveillance, stage distributions of CRC with and without surveillance, and five-year survival rates of CRC. General death rates at different age groups, which were obtained from the Department of Household Registration of the Taiwan government, were also incorporated in the Markov model to account for deaths of the relatives other than CRC. Same as the assumptions in other studies [9, 13], we assumed that an individual developed CRC at most two times in his or her lifetime. Moreover, an individual was assumed to recover from either the first or second CRC if the individual had survived for more than 10 years [13]. LYs were discounted at the 3% rate. The decision analytic model was implemented with the statistical language R. To obtain stable estimates of LYs and costs, the experiments were performed 1,000 times and the averaged estimates over 1,000 replicates were obtained.

### Sensitivity analyses

We performed probabilistic sensitivity analyses to account for the uncertainty of the input parameters. The theoretical distributions of the input variables were similar to those used in Ladabaum et al. [10] and Severin et al. [13]. That is, beta distributions were assumed for probabilities, gamma distributions were assumed for costs, log-normal distributions were assumed for reduced risks, and Poisson distribution was assumed for the number of relatives. The parameters of the theoretical distributions can also be found in S1 Table. A total of 1,000 replicates of the analyses based on the decision analytic model were performed. For each replicate, parameters values were randomly generated from the aforementioned distributions. Furthermore, we performed a one-way sensitivity analysis for Strategy 1. The upper and lower bounds for each input parameter were also shown in S1 Table.

## Results

Table 2 shows the numbers of LS probands in patients newly diagnosed with CRC, the numbers of relatives tested for LS, the numbers of relatives with LS mutations detected, and the costs of screening for different testing strategies. The total number of relatives with LS mutations was 743 in the simulated population. As expected, Strategy 4 was the most efficient strategy to identify relatives with LS mutations. Strategy 4 identified 28.9% of relatives with LS mutations, followed by 25%, 24%, and 23.8% for Strategies 3, 2, and 1, respectively. However, Strategy 4 was also the most expensive strategy, followed by Strategies 3, 2, and 1. Strategy 4 was approximately 9 times more expensive than Strategy 1 in terms of the total costs, compared to 5 times in Mvundura et al. [9]. This reflects the fact that sequencing is significantly more expensive than MSI and IHC analyses in Taiwan, when compared with costs in US as used in Mvundura et al. [9]. For example, the costs of sequencing a gene were 8.5 and 1.7 times higher than the costs of MSI analysis in our study and that in Mvundura et al. [9], respectively.

**Table 2:** Numbers of LS probands and relatives and costs for the four strategies.

	Strategy 1	Strategy 2	Strategy 3	Strategy 4
No. of LS probands	176	177	184	259
No. of relatives tested for LS	366	369	384	539
No. of relatives with LS mutations detected	177	178	186	215
Cost of detecting LS in newly diagnosed patients with CRC	$1,498,891	$1,731,350	$5,364,843	$37,365,594
Cost of detecting LS in relatives	$44,121	$45,210	$56,703	$65,356
Cost of surveillance and treatment for CRC for relatives with LS mutations	$2,716,955	$2,712,672	$2,909,862	$2,666,703
Total costs	$4,259,967	$4,489,232	$8,331,408	$40,097,654

Table 3 shows the discounted LYs and discounted costs per relative with LS mutations, and incremental costs per LY gained (i.e., incremental cost-effectiveness ratios or ICERs). As can be seen, Strategies 1 to 4 all increased the LYs per person relative to the Referent strategy; however, they were costlier than the Referent strategy. The ICERs were calculated with respect to the Referent strategy and to the next most cost-effective strategy in the remaining strategies. The ICERs with respect to the Referent strategy ranged from $6,025 for Strategy 1 to $145,110 for Strategy 4, whereas ICERs with respect to the next most cost-effective strategy ranged from $6,025 for Strategy 1 to $988,217 for Strategy 4.

**Table 3:** Cost-effectiveness analysis results based on ICER among different strategies.

Strategy	Discounted LYs per person	Discounted cost per person	Incremental costs per LY gained (relative to Referent strategy)	Incremental costs per LY gained (relative to the previous strategy)
Referent	21.551	$4,032		
1	21.834	$5,735	$6,025	$6,025
2	21.835	$6,044	$7,088	$260,824
3	21.852	$11,217	$23,872	$302,129
4	21.895	$53,985	$145,110	$988,217

**Fig 2:**
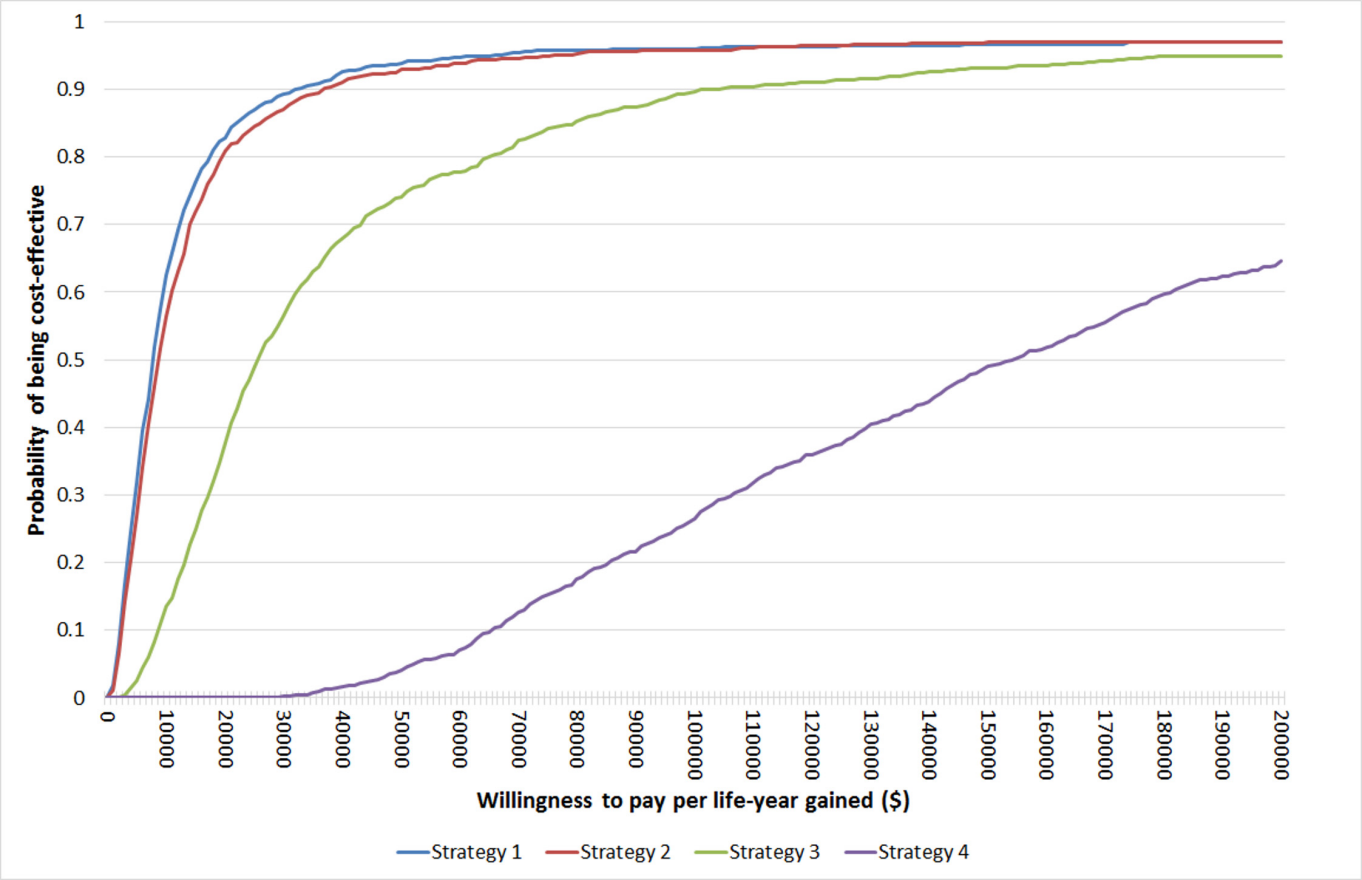
Cost-effectiveness acceptability curves for Strategies 1–4 relative to the Referent strategy.

Fig 2 shows the cost-effectiveness acceptability curves for Strategies 1–4 relative to the Referent strategy. At a commonly used threshold of $50,000 per life-year gained (LYG) to determine whether a strategy is cost-effective, Strategies 1, 2, 3 had probabilities of 0.939, 0.929, and 0.741, respectively, of being cost-effective relative to the Referent strategy, whereas Strategy 4 had a probability of only 0.041 of being cost-effective. At a lower threshold of $25,000 per LYG, Strategies 1 and 2 also had high probabilities (i.e., 0.871 and 0.846, respectively) of being cost-effective relative to the Referent strategy, whereas Strategy 3 had a probability of 0.491 and Strategy 4 had a probability close to 0 of being cost-effective.

**Fig 3:**
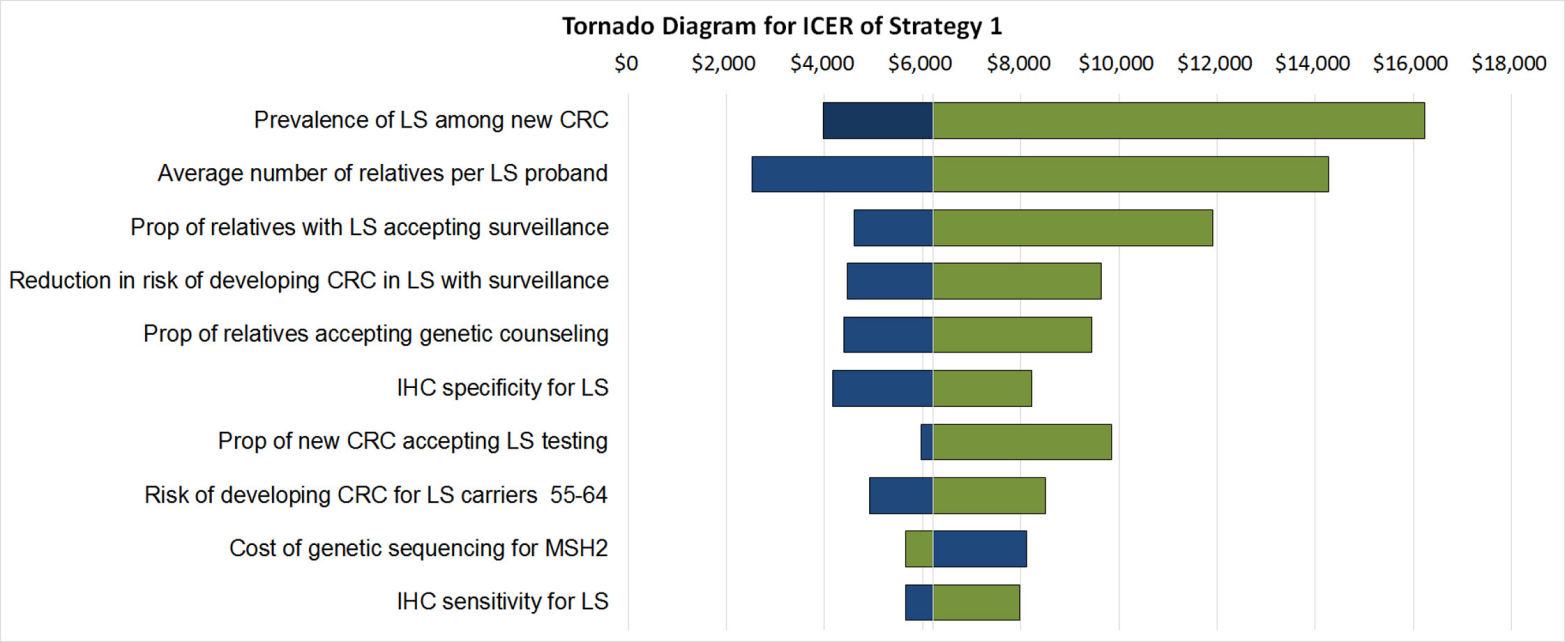
One-way sensitivity analysis results for Strategy 1. The blue and green bars represent the changes of ICER using upper and lower bound values, respectively, in S1 Table.

Fig 3 shows the one-way sensitivity analysis result using a tornado diagram for Strategy 1 relative to the Referent strategy. Note that the costs provided by NHI were considered as fixed values in the analysis. The most influential variable in ICER is the prevalence of LS among patients newly diagnosed with CRC, followed by the average number of relatives contacted for testing per LS proband, the proportion of relatives with LS mutations accepting increased surveillance, the reduction in risk of developing CRC in LS relatives with surveillance, and the proportion of relatives accepting genetic counseling. In the sensitivity analysis results in Mvundura et al. [9], the most influential variable is the risk of developing CRC among relatives. However, the influence of the variable decreased in their model using the more conservative parameters in Grosse et al. [16] (personal communication with Dr. Grosse). When excluding the variable of the risk of developing CRC among relatives in the sensitivity analysis results in Mvundura et al. [9], the five most influential variables in our analysis were the same as those reported by Mvundura et al. [9], regardless of the rank of the variables.

## Discussion

In this study, we adopted the four screening strategies used in Mvundura et al. [9] to evaluate the cost-effectiveness of the screening strategies for LS among relatives of patients newly diagnosed with CRC, while the population-specific parameters for Taiwan were used. The most cost-effective strategy is Strategy 1, with an ICER of $6,025 relative to the Referent strategy, whereas the most expensive strategy is Strategy 4, with an ICER of $988,217 relative to Strategy 3.

Taiwan’s gross domestic product (GDP) per capita was $21,308 in 2012. The World Health Organization (WHO) defines three categories of cost-effectiveness: highly cost-effective when ICER < GDP per capita, cost-effective when ICER is between one and three times GDP per capita, and not cost-effective when ICER is higher than three times the GDP per capita [25]. Using the WHO standard, Strategy 1 is considered as highly cost-effective relative to the Referent strategy, while Strategies 2–4 would not be considered as cost-effective relative to the previous strategy. The conclusions were consistent with other studies [9, 10, 16] that IHC testing followed by BRAF testing is the most cost-effective approach in all of the strategies evaluated when excluding the clinical criteria. The results will be informative to the Ministry of Health and Welfare of the Taiwan government when considering the implementation of a screening strategy for LS in Taiwan.

Several population-specific parameters, such as mutation rates in MMR genes and costs of genetic testing, can result in the difference in the analysis results between our study and others. For example, our results show that Strategy 4 has a significantly higher cost than that of Strategy 1, compared with the results in Mvundura et al. [9]. This finding can be attributed to the higher cost of sequencing in Taiwan than that in the US. With the rapid development of next-generation sequencing (NGS) technology, we expect the cost of Strategy 4 to be reduced significantly over the coming years. In relation to this development, cost-effectiveness analysis may be required routinely to evaluate these strategies with the latest parameter values. Also note that some costs in Taiwan, typically the costs based on the NHI reimbursements such as the costs of colonoscopy and IHC test, are relatively low by international standards. For example, the cost of colonoscopy is $76 in Taiwan when compared with $650-$700 in the US [9, 10]. Although the cost is low, it covers the costs of preparation, cleaning and disinfection of colonoscope as well as the cost of labor. The hospital medical service quality is regularly assessed so that the quality of disinfection and preservation for colonoscope is adequate.

In the cost-effectiveness analysis for the screening strategies by Severin et al. [13] based on the German data, the rate of uptake of genetic counseling and testing among relatives was lower than that in the US (i.e., 29.5% [26] used in their study compared with 52% used in Mvundura et al. [9]), thus contributing to a far higher cost for screening strategies in Germany. Our one-way sensitivity analysis also supported the finding that the rate of uptake of genetic counseling among relatives has a significant impact on the ICER (ranked as the 5^th^ most influential parameter in ICER of Strategy 1). The rate is unknown in Taiwan, because genetic screening for LS among patients newly diagnosed with CRC is not implemented by NHI and generally not offered by hospitals. Further studies are required to estimate the rate in Taiwan. Moreover, as suggested by Severin et al. [13], factors that motivate relatives to participate in genetic testing should be considered when implementing a genetic screening program for LS

Meanwhile, some limitations of the analysis as discussed in Mvundura et al. [9] are also applicable to our study. For example, the cost-effectiveness of the testing strategies relative to the use of Amsterdam or Bethesda family history criteria was not considered in this study because accurately obtaining family history information may be difficult and expensive. Moreover, lifetime costs of treatments for the second CRC have not been estimated in Taiwan. The costs can be calculated by using a strategy similar to that used in Chen et al. [17] using the NHI health insurance database in Taiwan. Furthermore, in this study, the risks of developing CRC in relatives with LS mutations for different age groups were adopted from a study in France based on 537 families carrying LS mutations [4], due to the fact that such information regarding the Chinese population is lacking in the literature. Owing to genetic heterogeneity and the difference in diet and lifestyle factors between the Taiwan and French populations, the risks may also be different. Therefore, further evaluations of the cost-effectiveness of different screening strategies for LS are required when such parameter values are available.

Our analysis was performed based on the NHI perspective. It is also important to evaluate the cost-effectiveness of testing strategies from the perspective of relatives with LS mutations. For example, when none of the four testing strategies are offered by NHI, relatives of patients newly diagnosed with CRC might wish to adopt a genetic testing with the costs being either self-paid or reimbursed by insurance policies purchased through private insurance companies. The results of the analysis based on the perspective of relatives with LS mutations will enable them to select the appropriate screening strategy based on their budget or insurance coverage.

In conclusion, Strategy 1 (IHC test followed by BRAF test) evaluated in our study has been found to be cost-effective relative to the Referent strategy. This is the first cost-effectiveness analysis for evaluating different genetic testing strategies for LS in Taiwan. As such, the results will be informative for the NHI when considering offering screening for LS in patients newly diagnosed with CRC.

## Supporting Information

S1 Table Parameter values and distributions used in the analysis. (DOCX)
