# Supplementary material for: Cost-Effectiveness Analysis of Different Genetic Testing Strategies for Lynch Syndrome in Taiwan
Source: PLoS One. 2016 Aug 2;11(8):e0160599. doi: 10.1371/journal.pone.0160599 (PMC4970721; doi:10.1371/journal.pone.0160599)
Supplement: S1 Table — (DOCX) [file pone.0160599.s001.docx]

S1 Table. Parameter values used in the analysis.

| Variable description | Base | Source | Range in sensitivity analysis | Theoretical distribution for sensitivity analysis |
| --- | --- | --- | --- | --- |
| Number of newly diagnosed patients with CRC | 13,534 | CRAR^a^ | Fixed | Fixed |
| Proportion of newly diagnosed patients with CRC accepting LS testing | 67% | [1,2] | 23-97% | Beta(5.9,2.9) |
| Prevalence of LS among newly diagnosed patients with CRC | 2.3% | [3] | 1.06-3.54% | Beta(12.8,547.1) |
| Proportion of LS with *MSH2* mutation | 25% | See Table 1 | 17-33% | Beta(77.5,232.5) |
| Proportion of LS with *MLH1* mutation | 58% | See Table 1 | 39-77% | Beta(179.8,130.2) |
| Proportion of LS with *MSH6* mutation | 11% | See Table 1 | 8-14% | Beta(34.1,275.9) |
| Proportion of LS with *PMS2* mutation | 6% | See Table 1 | 4-8% | Beta(18.6,291.4) |
| FIT sensitivity for CRC | 81.5% | [4] | 70.2-89.2% | Beta(52.1,11.8) |
| Sequencing sensitivity for LS | 99.5% | [1,2] | 98.6-99.9% | Beta(0.38,0.002) |
| Sequencing (1-sensitivity) for LS | 0.04% | [1,2] | 0.005-0.1% | Beta(0.006,14.9) |
| MSI sensitivity for *MLH1/MSH2* mutations | 89% | [1,2] | 81-95% | Beta(23.3,2.8) |
| MSI sensitivity for *MSH6/PMS2* mutations | 76% | [1,2] | 54-91% | Beta(37.7,11.9) |
| MSI specificity for LS | 90.2% | [1,2] | 85-94% | Beta(21.2,2.3) |
| IHC sensitivity for LS | 83% | [1,2] | 63-96% | Beta(31.7,6.4) |
| IHC specificity for LS | 88.8% | [1,2] | 83-94% | Beta(23.6,2.9) |
| BRAF sensitivity for IHC *MLH1* | 69% | [1,2] | 50-85% | Beta(40.3,18.1) |
| BRAF specificity for IHC *MLH1* | 99% | [1,2] | 98-99.7% | Beta(1.7,0.017) |
| Average number of relatives per LS proband | 4 | [1,2] | 2-8 | Poisson(4) |
| Proportion of first-degree relatives with a mutation | 50% |  | fixed | fixed |
| Proportion of relatives accepting genetic counseling | 52% | [1,2] | 34-70% | Beta(17.9,16.5) |
| Proportion of relatives with LS accepting increased surveillance | 79% | [1,2] | 46-98% | Beta(526.7,130.2) |
| Risk of perforation during colonoscopy | 0.11% | [1,2] | 0.08-0.15% | Beta(1.1,997) |
| Risk of bleeding during colonoscopy | 0.33% | [1,2] | 0.23-0.43% | Beta(3.3,995) |
| Risk of dying from a colonoscopy | 0.008% | [1,2] | 0.006-0.01% | Beta(0.08,998) |
| Risk of developing CRC for LS carriers between 25 and 34 | 0.2% | [5] | 0.05-0.35% | Beta(0.0007,34.2) |
| Risk of developing CRC for LS carriers between 35 and 44 | 0.3% | [5] | 0.07-0.53% | Beta(7,3475) |
| Risk of developing CRC for LS carriers between 45 and 54 | 0.8% | [5] | 0.14-1.46% | Beta(6.6,2215) |
| Risk of developing CRC for LS carriers between 55 and 64 | 1.1% | [5] | 0.1-2.1% | Beta(5.8,722) |
| Risk of developing CRC for LS carriers between 65 and 74 | 1.1% | [5] | 0.1-2.1% | Beta(4.8,429) |
| Risk of developing CRC for LS carriers > 74 | 0.7% | [5] | 0.1-1.3% | Beta(4.8,429) |
| Risk of second CRC diagnosis for LS carriers | 1.6% | [1,2,6] | 0.3-2.9% | Beta(5.9,367) |
| Reduction in risk of developing CRC | 59% | [7,8] | 35-80% | Log-normal(-1.1,0.62) |
| Stage distribution of CRC at diagnosis without surveillance |  |  |  |  |
| Localized—Stage 1 | 21.2% | [9] | fixed | fixed |
| Regional—Stage 2 | 27.8% | [9] | fixed | fixed |
| Distant—Stage 3 | 31.8% | [9] | fixed | fixed |
| Unstaged—Stage 4 | 19.2% | [9] | fixed | fixed |
| Stage distribution of CRC at diagnosis with FIT |  |  |  |  |
| Localized—Stage 1 | 48.2% | [9] | fixed | fixed |
| Regional—Stage 2 | 20.9% | [9] | fixed | fixed |
| Distant—Stage 3 | 23.7% | [9] | fixed | fixed |
| Unstaged—Stage 4 | 7.2% | [9] | fixed | fixed |
| Stage distribution of CRC at diagnosis with surveillance |  |  |  |  |
| Localized—Stage 1 | 72% | See manuscript | fixed | fixed |
| Regional—Stage 2 | 14% | See manuscript | fixed | fixed |
| Distant—Stage 3 | 12% | See manuscript | fixed | fixed |
| Unstaged—Stage 4 | 2% | See manuscript | fixed | fixed |
| Five-year relative survival rate for CRC by stage at diagnosis |  |  |  |  |
| Localized—Stage 1 | 88% | [10] | fixed | fixed |
| Regional—Stage 2 | 77% | [10] | fixed | fixed |
| Distant—Stage 3 | 62% | [10] | fixed | fixed |
| Unstaged—Stage 4 | 14% | [10] | fixed | fixed |
| Cost of iFOBT | $6.66 | NHI^b^ | fixed | fixed |
| Cost of initial counseling before genetic sequencing | $16.66 | NHI | fixed | fixed |
| Costs of genetic sequencing |  |  |  |  |
| MLH1 | $999.6 | SP^c^ | $833-$1,332 | Gamma(144,0.144) |
| MSH2 | $999.6 | SP | $833-$1,332 | Gamma(144,0.144) |
| MSH6 | $999.6 | SP | $833-$1,332 | Gamma(144,0.144) |
| PMS2 | $999.6 | SP | $833-$1,332 | Gamma(144,0.144) |
| BRAF V600E | $99.96 | SP | $66.64-$133.28 | Gamma(9,0.09) |
| Cost of deletion analysis for *MSH6* and *PMS2* | $83.3 | SP | $66.64-$99.96 | Gamma(100,1.2) |
| Cost of post-test genetic counseling | $16.66 | NHI | fixed | fixed |
| Cost of information session before MSI or IHC testing | $16.66 | NHI | fixed | fixed |
| Cost of MSI analysis | $116.62 | SP | $66.64-$166.60 | Gamma(21.7,0.018) |
| Cost of IHC analysis | $45.12 | NHI | fixed | fixed |
| Cost of locating and approaching relative | $16.66 | EO^d^ | $6.66-$26.65 | Gamma(11.1,0.6) |
| Cost of sequencing for family mutation in relative | $49.98 | SP | $33.32-$66.64 | Gamma(36,0.72) |
| Cost of colonoscopy | $74.97 | NHI | fixed | fixed |
| Cost of treating perforation complication | $347.19 | NHI | fixed | fixed |
| Cost of treating bleeding complication | $359.86 | NHI | fixed | fixed |
| Treatment costs for CRC |  |  |  |  |
| First CRC diagnosed at Stage 1 | $8,416 | [10] | $6,477-$10,355 | Gamma(72.4,0.0086) |
| First CRC diagnosed at Stage 2 | $8,416 | [10] | $6,477-$10,355 | Gamma(72.4,0.0086) |
| First CRC diagnosed at Stage 3 | $14,334 | [10] | $12,579-$16,089 | Gamma(256,0.018) |
| First CRC diagnosed at Stage 4 | $21,837 | [10] | $20,139-$23,535 | Gamma(635,0.029) |
| Second CRC diagnosed at Stage 1 | $8,626 | See manuscript | $6,639-$10,614 | Gamma(72.5,0.0084) |
| Second CRC diagnosed at Stage 2 | $8,626 | See manuscript | $6,639-$10,614 | Gamma(72.5,0.0084) |
| Second CRC diagnosed at Stage 3 | $15,120 | See manuscript | $12,894-$16,492 | Gamma(177.4,0.012) |
| Second CRC diagnosed at Stage 4 | $23,741 | See manuscript | $20,643-$24,124 | Gamma(225.8,0.0095) |

^a^CRAR: cancer registry annual report published by the Ministry of Health and Welfare of the Taiwan government.

^b^NHI: the amount of reimbursement issued by thfe National Health Insurance Administration of the Taiwan government

^c^SP: self-paid cost reported by the 21 major hospitals in Taiwan

^d^EO: expert opinion

**References**

1. Mvundura M, Grosse SD, Hampel H, Palomaki GE (2010) The cost-effectiveness of genetic testing strategies for Lynch syndrome among newly diagnosed patients with colorectal cancer. Genet Med 12: 93-104.

2. Palomaki GE, McClain MR, Melillo S, Hampel HL, Thibodeau SN (2009) EGAPP supplementary evidence review: DNA testing strategies aimed at reducing morbidity and mortality from Lynch syndrome. Genet Med 11: 42-65.

3. Chang SC, Lin PC, Yang SH, Wang HS, Liang WY, et al. (2010) Taiwan hospital-based detection of Lynch syndrome distinguishes 2 types of microsatellite instabilities in colorectal cancers. Surgery 147: 720-728.

4. Chen LS, Liao CS, Chang SH, Lai HC, Chen TH (2007) Cost-effectiveness analysis for determining optimal cut-off of immunochemical faecal occult blood test for population-based colorectal cancer screening (KCIS 16). J Med Screen 14: 191-199.

5. Bonadona V, Bonaiti B, Olschwang S, Grandjouan S, Huiart L, et al. (2011) Cancer risks associated with germline mutations in MLH1, MSH2, and MSH6 genes in Lynch syndrome. JAMA 305: 2304-2310.

6. Parry S, Win AK, Parry B, Macrae FA, Gurrin LC, et al. (2011) Metachronous colorectal cancer risk for mismatch repair gene mutation carriers: the advantage of more extensive colon surgery. Gut 60: 950-957.

7. Grosse SD, Palomaki GE, Mvundura M, Hampel H (2015) The cost-effectiveness of routine testing for Lynch syndrome in newly diagnosed patients with colorectal cancer in the United States: corrected estimates. Genet Med 17: 510-511.

8. Stupart DA, Goldberg PA, Algar U, Ramesar R (2009) Cancer risk in a cohort of subjects carrying a single mismatch repair gene mutation. Fam Cancer 8: 519-523.

9. Chiu HM, Chen SL, Yen AM, Chiu SY, Fann JC, et al. (2015) Effectiveness of fecal immunochemical testing in reducing colorectal cancer mortality from the One Million Taiwanese Screening Program. Cancer 121: 3221-3229.

10. Chen PC, Lee JC, Wang JD (2015) Estimation of Life-Year Loss and Lifetime Costs for Different Stages of Colon Adenocarcinoma in Taiwan. PLoS One 10: e0133755.
